# Stable Isotope Tracer Analysis in Isolated Mitochondria from Mammalian Systems

**DOI:** 10.3390/metabo4020166

**Published:** 2014-04-10

**Authors:** Simon-Pierre Gravel, Sylvia Andrzejewski, Daina Avizonis, Julie St-Pierre

**Affiliations:** 1 Rosalind and Morris Goodman Cancer Research Centre, 1160 Pine Ave. West, Montréal H3A 1A3, Québec, Canada; E-Mails: simon-pierre.gravel@mcgill.ca (S.-P.G.); sylvia.andrzejewski@mail.mcgill.ca (S.A.); daina.avizonis@mcgill.ca (D.A.); 2 Department of Biochemistry, McGill University, McIntyre Building, 3655 prom. Sir William Osler, Montréal H3G 1Y6, Québec, Canada

**Keywords:** mitochondria, muscle, cells, stable isotope tracer analysis, citric acid cycle, rotenone, antimycin, Neu/ErbB2, cancer, GC/MS

## Abstract

Mitochondria are a focal point in metabolism, given that they play fundamental roles in catabolic, as well as anabolic reactions. Alterations in mitochondrial functions are often studied in whole cells, and metabolomics experiments using ^13^C-labeled substrates, coupled with mass isotopomer distribution analyses, represent a powerful approach to study global changes in cellular metabolic activities. However, little is known regarding the assessment of metabolic activities in isolated mitochondria using this technology. Studies on isolated mitochondria permit the evaluation of whether changes in cellular metabolic activities are due to modifications in the intrinsic properties of the mitochondria. Here, we present a streamlined approach to accurately determine ^13^C, as well as ^12^C enrichments in isolated mitochondria from mammalian tissues or cultured cells by GC/MS. We demonstrate the relevance of this experimental approach by assessing the effects of drugs perturbing mitochondrial functions on the mass isotopomer enrichment of metabolic intermediates. Furthermore, we investigate ^13^C and ^12^C enrichments in mitochondria isolated from cancer cells given the emerging role of metabolic alterations in supporting tumor growth. This original method will provide a very sensitive tool to perform metabolomics studies on isolated mitochondria.

## 1. Introduction

Mitochondria are organelles that are well characterized for their role in cellular respiration, namely for coupling the reduction of molecular oxygen (O_2_) to ATP production. It is also important to appreciate that mitochondria are the host of other key metabolic pathways, such as the citric acid cycle (CAC), β-oxidation, urea cycle and heme biosynthesis. Mitochondria express specialized transporters that mediate the shuttle of metabolites in and out of the mitochondria [1], and thus, their global function is closely linked to the needs and uses of their cellular environment. Given the central role of mitochondria in metabolism, it is perhaps not surprising that altered mitochondrial metabolism is seen in numerous physiological and pathological conditions. Indeed, metabolic signatures of mitochondrial defects are starting to emerge in aging and neurodegeneration [2], as well as in cancer [3,4], and these findings are of interest for the diagnosis and treatment of mitochondrial diseases. Although evaluation of the steady-state level of metabolites can be very instructive, it does not provide much information regarding the dynamics of active metabolic pathways. The steady-state level of any given metabolite is dependent on its participation in defined metabolic pathway(s). The use of isotopes, such as ^13^C and ^15^N, is required to assess which metabolic pathways are actively contributing to the pool, as well as the directionality of active pathways [5,6,7]. ^13^C-glucose along with ^13^C and ^15^N-glutamine are examples of tracers that are routinely used to assess specific metabolic activities in mammalian cells [8,9].

Stable isotope tracer analyses in isolated mitochondria have only been done sporadically (see Table 1). The prevailing approach for analyzing mitochondrial ^13^C enrichments has been by NMR, with the manifest advantage of positional isotopic analysis. However, NMR has lower sensitivity compared to GC/MS or LC/MS and thus, in practice, cannot be easily used to assess low levels of CAC metabolites, such as oxaloacetate, isocitrate and cis-aconitate extracted from organelles [10]. Some of these limitations in sensitivity can be overcome through the use of hyperpolarized tracer metabolites; however, this technique requires instrumentation that is not generally available in most NMR facilities, and even so, metabolite coverage is still limited [11,12,13]. To address this, GC/MS-based methods were specifically developed to improve the sensitivity and coverage of CAC intermediates [14]. Here, we show that the application of GC/MS and mass isotopomer distribution analysis can be used to study CAC functions in isolated mitochondria from mammalian systems. This experimental approach has been applied to mitochondria isolated from mammalian tissues, as well as cancer cells, and has allowed us to compare these results with recent findings obtained from cell lines, in addition to highlighting novel observations.

## 2. Results and Discussion

### 2.1. Method Rationale and Assessment of Mitochondrial Functions

Metabolomics is a relatively recent “omics” science that has been applied successfully to cell cultures [14], animal and human tissues [15], fluids [16,17,18], micro-organisms [19,20] and model organisms [21]

**Table 1:** Publications performing ^13^C tracer analysis in isolated mitochondria.

Source	RCR	^13^C-substrate(s)	Incubation time	Analyzed Metabolites	Platform	Reference
Rat brain	³5	U-^13^C-glutamate	4 min	KG, Succ, Mal	NMR	[22]
Potato tubers	N/A	Multiple forms of ^13^C-glutamate	0–11 h	KG, Gln, Mal, Asp	NMR	[23]
*Arabidopsis* cells	N/A	1-^13^C-glucose	16 h	Glycolytic, Cit, Succ, Fum, Mal and others	GC/MS	[24]
		1-^13^C-fructose 1,6-bisphosphate				
*Arabidopsis* cells	N/A	1-^13^C-glucose	0–16 h	Glycolytic, Cit, Succ, Mal	NMR	[25]
		1-^13^C-fructose 1,6-bisphosphate				
Rat brain	3.0–4.5	U-^13^C-glutamate	5 min	Glu, Gln, Asp	LC/MS	[26]
		U-^13^C-glutamine				
		U-^13^C-succinate				

RCR, respiratory control ratio; N/A, unavailable information; KG, alpha-ketoglutarate; Succ, succinate; Mal, malate; Asp, aspartate; Glu, glutamate; Gln, glutamine; Glycolytic, glycolytic intermediates; Cit, citrate; NMR, nuclear magnetic resonance; GC, gas chromatography; LC, liquid chromatography; MS, mass spectrometry.

The steady-state concentration of metabolites can be obtained from multiple platforms, such as nuclear magnetic resonance, and liquid or gas chromatography coupled with mass spectrometry. In addition, these platforms can be used combinatorially to cover a wide range of metabolites. While proteomics [27] and lipidomics [28,29] studies have been applied to isolated mitochondria, few metabolomics studies have been performed on these organelles (Table 1). In order to measure CAC activity in isolated mitochondria, which is intimately linked to mitochondrial respiration, we developed a method that allows for the measurement of both respiratory capacities using a Clark-type electrode, as well as CAC activity using ^13^C-labeled and unlabeled metabolites. Such a dual analysis is expected to provide valuable information on highly specific mitochondrial remodeling mechanisms that occur in physiological and pathological conditions.

**Figure 1:**
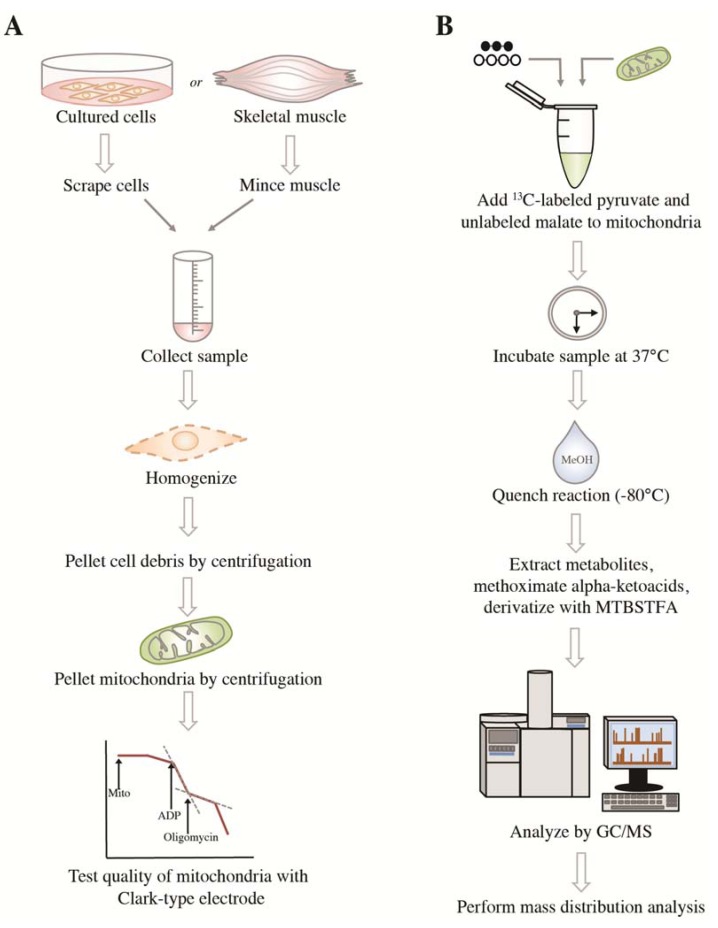
Schematic of experimental steps for stable isotope tracer analysis on isolated mitochondria. (**A**) Extraction of mitochondria, purification and assessment of quality. (**B**) Incubation of a fixed amount of mitochondria (evaluated by protein content) with substrates, such as ^13^C-pyruvate and unlabeled malate, sample preparation and GC/MS analysis. Complete methods are described in Section 3. MTBSTFA, N-Methyl-N-tert-butyldimethylsilyltrifluoroacetamide.

The complete experimental procedure presented in this article is depicted in Figure 1. We isolated mitochondria from murine skeletal muscle and cultured cells. The quality of the mitochondrial suspensions was evaluated by calculating respiratory control ratios (RCRs), which represent the ratio of State 3 (respiration in the presence of ADP and substrates) to State 4 (respiration in the presence of oligomycin) respiration rates. RCR values provide valuable information on the membrane integrity of isolated mitochondria [30] and thus, the quality of mitochondrial suspensions. We only analyzed mitochondrial suspensions that displayed RCR values above 3. Metabolic reactions were carried out at 37 °C for no longer than 30 min and quenched with cold methanol kept on dry ice. Metabolic reactions must be quenched rapidly and efficiently in order to eliminate any residual enzymatic activity that could affect metabolite composition and abundance.

After ^13^C pulse incubations, mitochondrial suspensions (mitochondria in assay medium) were immediately quenched with cold methanol, to minimize metabolite loss during further manipulations. Following extraction, metabolites were derivatized using an optimized procedure to limit potential degradation [31]. First, alpha-ketoacids were methoximated to stabilize known labile species, such as pyruvate, alpha-ketoglutarate and oxaloacetate [14]. Second, samples were derivatized with MTBSTFA (N-Methyl-N-tert-butyldimethylsilyltrifluoroacetamide) to produce volatile compounds appropriate for GC/MS analyses.

**Figure 2:**
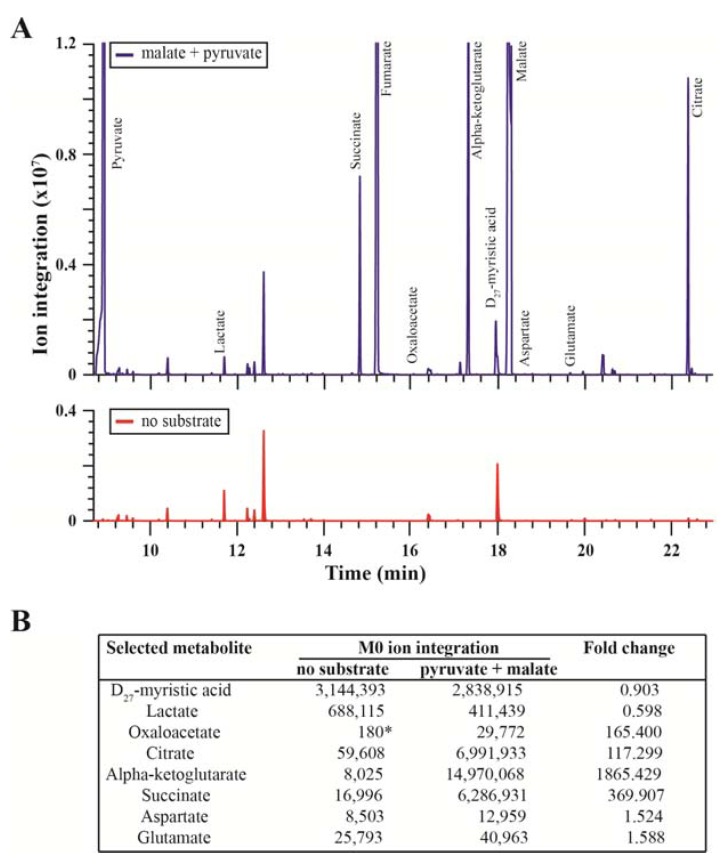
The selected metabolite profile of mitochondria isolated from mouse skeletal muscle obtained by GC/MS (selective ion monitoring (SIM) mode). (**A**) Representative chromatograms obtained from mitochondria incubated in the presence (upper panel, in blue) or absence (lower panel, in red) of respiratory substrates (1 mM pyruvate and 1 mM malate) for 15 minutes. Oxaloacetate, aspartate and glutamate are not abundant enough to be observed on the chromatograms at the depicted scale. The y-axis is set to 1.2 × 10^7^ (upper graph) and to 0.4 × 10^7^ (lower graph) to allow for better visualization of principal ion peaks. Pyruvate, fumarate, alpha-ketoglutarate and malate peaks are truncated. (**B**) M0 ion integrations for the metabolites studied in this article. The values represent the integrations obtained from the representative chromatograms in Panel A. Fold change values represent the ratio of the M0 ion integration in the presence of pyruvate + malate divided by that in the absence of substrates. Fold change values show that oxaloacetate, citrate, alpha-ketoglutarate and succinate are the metabolites most responsive to the presence of substrates. Pyruvate, fumarate and malate values are saturated and above the upper limit of detection; thus, they are excluded from our analyses. * Oxaloacetate integration in the absence of substrate shows signal-to-noise (S/N) values of Approximately 2.1, while all other integrations display S/N values above 100.

We first assessed the impact of the addition of unlabeled substrates on the metabolite composition of mitochondria isolated from murine skeletal muscle. An amount of mitochondria corresponding to 0.15 mg of mitochondrial protein was diluted into a modified assay buffer (Section 3.5), which is commonly used for mitochondrial respiration experiments [32]. The level of phosphate was lowered exactly 25 fold, so that, first, the derivatization reactants would not be exhausted by high phosphate concentrations and, second, that the unreacted phosphoric acid would not damage the GC column. The levels of 4-(2-hydroxyethyl)-1-piperazineethanesulfonic acid (HEPES) and ethylene glycol tetraacetic acid (EGTA) were also lowered to limit the protection or derivatization reaction interference and reactant consumption. Mitochondria maintained in this modified assay buffer displayed similar RCR values to those obtained in regular medium (unpublished data), illustrating that the modified buffer does not impact mitochondrial integrity. Using targeted analyses by GC/MS, we were able to assess the levels of CAC intermediates, as well as amino acids and lactate in isolated mitochondria, by the integration of m + 0 (M0) ion intensities. In order to determine the impact of respiratory substrates on mitochondrial metabolism, we incubated mitochondria for 15 minutes in the presence of unlabeled malate (1 mM) and U-^13^C-pyruvate (1 mM), a condition commonly known as State 2 respiration (baseline respiration) [33,34]. Comparison of basal and State 2 selected ion chromatograms obtained with selective ion monitoring (SIM) mode acquisition shows that the addition of substrates resulted in a dramatic increase in malate and pyruvate levels, along with many other metabolites, such as succinate, fumarate, alpha-ketoglutarate and citrate (Figure 2A,B). Other metabolites, such as oxaloacetate, presenting very low abundance in mitochondria in the absence of substrates, also showed strong enrichment upon incubation with respiratory substrates, while lactate, glutamate and aspartate were less affected. These results show that mitochondria incubated with substrates exhibit quick and strong changes in CAC intermediate levels, thus validating our experimental design for stable isotope tracer analysis. It is also important to note that this simple experimental approach can be used for untargeted analysis using scan mode acquisitions with isolated mitochondria. Here, we used a more targeted (SIM) approach to help enhance the signal of metabolites exhibiting a low concentration.

### 2.2. Stable Isotope Tracer Analyses of Skeletal Muscle Mitochondria Treated with Electron Transport Chain (ETC) Inhibitors

Electron transport chain (ETC) inhibitors (or mitotoxins) target specific complexes within the mitochondrial ETC and perturb mitochondrial metabolism.

Rotenone, a Complex I inhibitor, blocks the usage of reductive equivalents provided by reduced nicotinamide adenine dinucleotide (NADH) leading to an accumulation of NADH [35]. This increase in NADH will consequently impact CAC activity. Antimycin A (AA) is a Complex III inhibitor, and inhibition of this complex will result in accumulation of upstream electron rich species, which in turn will impact Complexes I and II functions and consequently, CAC activity [32]. Thus, it is expected that the use of ETC inhibitors will impact CAC functions. Recent studies have shown changes in CAC intermediates in cultured cells treated with mitotoxins [14,36]. Although ETC inhibitors or mitochondrial dysfunction can modify the directionality of the CAC in live cells [8,37], it is unknown whether this biological phenomenon can be captured in isolated mitochondria. Moreover, it cannot be assumed that stable isotope enrichments or steady-state metabolite measurements obtained from cells reflects intrinsic mitochondrial behavior.

**Figure 3:**
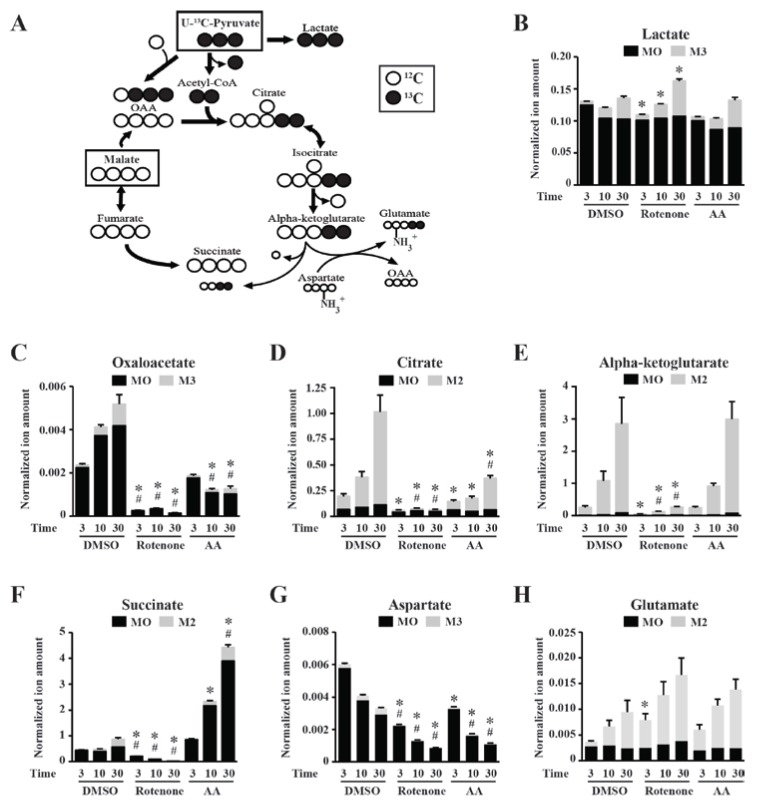
Stable isotope tracer analysis in skeletal muscle mitochondria treated with mitochondrial inhibitors and incubated with ^13^C-pyruvate and unlabeled malate as respiratory substrates. (**A**) Diagram depicting carbon cycling into the citric acid cycle (CAC). Substrates used during mitochondrial incubations are shown in boxes. ^13^C labeling is black and ^12^C is white. Enzymes, co-substrates and cofactors are not shown. (**B–F**) Control dimethyl sulfoxide (DMSO), rotenone and antimycin A (AA) treatments of isolated murine skeletal muscle mitochondria were performed for the indicated times in minutes, in the presence of U-^13^C-pyruvate and unlabeled malate. Isotopic enrichment for lactate (**B**), oxaloacetate (**C**), citrate (**D**), alpha-ketoglutarate (**E**), succinate (**F**), aspartate (**G**) and glutamate (**H**) are shown as the means of m + 0 (M0) superimposed with the means of m + 2 (M2) or m + 3 (M3) enrichments ± SEM of 4 independent experiments. * *p* < 0.05, one-way repeated measures ANOVA, followed by a Dunnett’s test, performed on fold differences in isotopomer enrichments (M2 or M3) *versus* the DMSO control for each specific time point. # *p* < 0.05, one-way repeated measures ANOVA, followed by a Dunnett’s test, performed on fold differences in M0 *versus* the DMSO control for each specific time point. “Normalized ion amount” represents the values obtained for the mass isotopomer distribution (MID) × corrected area (Section 3.7).

In order to assess the impact of ETC inhibitors on mitochondrial CAC activity, mitochondria isolated from murine skeletal muscle were incubated with 1 mM of U-^13^C-pyruvate (uniformly ^13^C-labeled pyruvate) and 1 mM of unlabeled malate. Labeled pyruvate was used to assess its specific contribution and directionality inside the CAC (Figure 3A). Mitochondrial suspensions were quenched following incubation times of 3, 10 and 30 minutes. To evaluate the isotopic enrichment of metabolites generated upon incubation with respiratory substrates, we performed mass isotopomer distribution analyses (described in Section 3.7). As shown in Figure 3C–F, both the amount and isotopic enrichment of multiple CAC intermediates obtained from control dimethyl sulfoxide (DMSO)-treated mitochondria increased with incubation time. Citrate m + 2 and alpha-ketoglutarate m + 2 showed very similar enrichment over time, while succinate m + 2 was poorly labeled throughout the incubation period. Interestingly, oxaloacetate (OAA) displayed an increase in both the unlabeled (m + 0) and labeled (m + 3) fractions (Figure 3C). m + 0 enrichment could be explained by a direct conversion of the exogenous unlabeled malate to OAA or the conversion of endogenous aspartate to OAA via the transaminase, GOT2 (Figure 3G). Glutamate m + 2 enrichment supports this point of view (Figure 3H). The m + 3 species of OAA are derived from U-^13^C-pyruvate following a carboxylation step catalyzed by pyruvate carboxylase, a mitochondrial protein known to be expressed in skeletal muscle [38] (Figure 3A). We also detected small enrichments in lactate m + 3 (Figure 3B). This finding supports the reported close association of lactate dehydrogenase (LDH) to mitochondria, which converts pyruvate to lactate [39].

The experimental design to test the impact of mitochondrial inhibitors was such that the inhibitors were given to the mitochondrial suspensions immediately after substrate addition, so full inhibition of a specific ETC target was not necessarily achieved at the onset of the time course. The ETC inhibitors, rotenone and AA, caused striking changes in mitochondrial carbon flow compared with the control treatment. Although rotenone completely abolished the detectable formation of citrate m + 2 (Figure 3D), a small fraction of alpha-ketoglutarate m + 2 was detected (Figure 3E), suggesting that the citrate m + 2 generated prior to full inhibition by rotenone is rapidly converted to alpha-ketoglutarate m + 2 and does not accumulate. The citrate generated before complete inhibition is expected to accumulate into alpha-ketoglutarate, the most highly enriched metabolite upon incubation with pyruvate and malate (Figure 2B).

The consistent tendency of mitochondrial suspensions to accumulate alpha-ketoglutarate could be explained by unbalanced reaction rates for the production and usage of this metabolite. In support of this point, there is a mismatch between alpha-ketoglutarate m + 2 and succinate m + 2 (Figure 3F), as well as glutamate m + 2 (Figure 3H). This imbalance can also be observed upon rotenone treatment when comparing glutamate m + 2 (Figure 3H) with succinate m + 2 (Figure 3F). Rotenone is expected to inhibit enzymes, such as pyruvate dehydrogenase, isocitrate dehydrogenase and alpha-ketoglutarate dehydrogenase, through NADH accumulation. Inhibition of the latter will allow alpha-ketoglutarate to use alternative routes, such as its transamination by aspartate via GOT2, and this point is supported by stronger m + 2 enrichment compared to the DMSO control (Figure 3H). Finally, the unlabeled fractions of OAA and succinate were reduced upon rotenone treatment, indicating that the unlabeled malate was not used efficiently for CAC replenishment. Treatments with AA led to less dramatic changes in CAC metabolite species. Indeed, citrate m + 2 was diminished, while alpha-ketoglutarate m + 2 was unaffected by AA treatment. This suggests that quantification of citrate alone is not sufficient to ensure complete understanding of CAC activity. AA had a pronounced effect on the m + 0 fraction of succinate (Figure 3F), despite the weaker effect on the enrichment of m + 2 species. Since AA will impair ETC activity upstream of its site of action in Complex III (see above), a likely explanation for the accumulation of succinate m + 0 is that it originates from unlabeled malate during reverse cycling. Finally, rotenone and AA treatment resulted in a significant increase in lactate m + 3 (Figure 3B), likely due to the fact that CAC is inhibited, causing U-^13^C-pyruvate to use alternative routes, such as its reduction to lactate. In agreement with the results obtained for CAC intermediates, AA caused smaller changes in lactate m + 3 than rotenone. In fact, the changes in lactate m + 3 upon mitotoxins exposure only reached statistical significance for rotenone (Figure 3B).

### 2.3. Stable Isotope Tracer Analyses in Mitochondria Isolated from Cancer Cells

**Figure 4:**
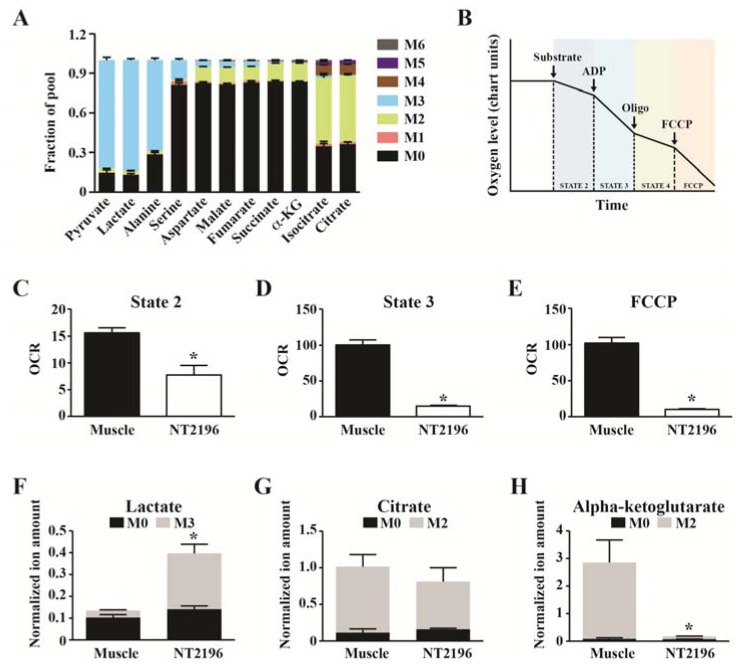
Stable isotope tracer analyses of cells and isolated mitochondria from mammalian systems. (**A**) Mass isotopomer analyses of metabolites extracted from NT2196 cells incubated with 10 mM U-^13^C-glucose for 1 h. M0 to M6 represent the m + 0 to m + 6 enrichments and are indicated by different colors. Data are shown as means ± SEM of five independent experiments. (**B**) The experimental design for respiration analyses in isolated mitochondria. The addition of substrates/drugs to isolated mitochondria is indicated by arrows above the trace. The addition of ADP stimulates ATP production. Oligomycin inhibits ATP synthase and thus, respiration coupled to ATP production, and FCCP (carbonyl cyanide-*4*-(trifluoromethoxy) phenylhydrazone) uncouples respiration from ATP production, representing the maximal respiratory capacity of mitochondria. (**C–E**) Comparison of State 2 (**C**), State 3 (**D**) or FCCP (**E**) rates in isolated mitochondria from murine skeletal muscle and NT2196 cells. Oxygen consumption rate (OCR) is the rate of oxygen consumption (chart units) normalized per minute per mg of mitochondrial protein, where 1 chart unit is 0.2% oxygen. Data are shown as means ± SEM of 4 independent experiments for NT2196 cells and 10 for muscle tissue. * *p* < 0.05, Student’s *t*-test. (**F–H**) Isotopic enrichment for lactate (**F**), citrate (**G**) and alpha-ketoglutarate (**H)** in isolated mitochondria from murine skeletal muscle and NT2196 cells incubated with U-^13^C-pyruvate and unlabeled malate for 30 minutes at 37 °C. Data are shown as means ± SEM of 4 independent experiments. * *p* < 0.05, Student’s *t*-test. “Normalized ion amount” is used to define the values obtained from the MID × corrected area (Section 3.7).

Cancer cells often display altered metabolism, notably an increased reliance on aerobic glycolysis to support ATP production, a biological phenomenon known as the Warburg effect [40]. ErbB2/HER2 receptor positive (HER2+) cancer cells have been shown to display the Warburg effect in parallel to decreased respiration [41,42]. Mammary epithelial cells expressing an oncogenic Neu receptor and derived from explants (NT2196) were used as a murine model of HER2+ breast cancer. These cells have been shown to exhibit the Warburg effect and decreased mitochondrial respiration [42,43]. In support of these reported changes in metabolism, NT2196 cells incubated with 10 mM U-^13^C-glucose display a strong labeling of pyruvate and lactate, indicating high glycolytic activity, along with minimal labeling of CAC metabolites, supporting reduced mitochondrial functions (Figure 4A). Strikingly, the fraction of alpha-ketoglutarate labeled is smaller than the one from citrate and isocitrate. This suggests that a significant part of the glucose-derived citrate pool could be used in these cells to support lipogenesis, while its contribution to oxidative phosphorylation might be smaller. In agreement with these observations, we have recently shown that HER2+ cancer cells are dependent on glutamine for proliferation and that glutamine is used to replenish the CAC during both forward and reverse cycling [44].

Changes in cellular respiration could be explained by alterations in mitochondrial density, modifications in the intrinsic properties of individual mitochondria or changes in substrate supply to the mitochondria. Very little work has been performed to evaluate the metabolic properties of mitochondria isolated from cancer cells. We assessed the basal (State 2), active (State 3) and maximal respiratory capacities (using carbonyl cyanide-*4*-(trifluoromethoxy)phenylhydrazone (FCCP)), as well as the CAC activity of mitochondria isolated from NT2196 cells (Figure 4B). We compared their mitochondrial activities to that of mitochondria isolated from murine skeletal muscle. The rationale for this comparison was that it would contrast mitochondria isolated from cells that rely minimally on mitochondria for ATP production (HER2+ cancer cells) with those from cells that rely mainly on mitochondria for ATP production (muscle).

Mitochondria from NT2196 cells displayed significantly lower basal, active and maximal respiratory capacities than those from muscle tissues (Figure 4C–E). The differences in respiratory capacities were particularly striking for the State 3 and FCCP conditions, where the mitochondria from NT2196 cells displayed respiration rates that were approximately one fifth of those from muscle mitochondria (Figure 4D–E). In order to determine CAC activity, isolated mitochondria from NT2196 cells and muscle tissues were incubated with U-^13^C-pyruvate (1 mM) and unlabeled malate (1 mM) for 30 min. We first analyzed the isotopic enrichments of citrate and alpha-ketoglutarate. Mitochondria from muscle tissues and NT2196 cells showed a similar enrichment of citrate m + 2, but a profound difference in that of alpha-ketoglutarate m + 2 (Figure 4G–H). This inability of mitochondria isolated from NT2196 cells to generate alpha-ketoglutarate m + 2 is fully concordant with the reduced ability of NT2196 cells to generate alpha-ketoglutarate m + 2 from U-^13^C-glucose (Figure 4A). Together, these results illustrate that mitochondria isolated from muscle tissues and cancer cells exhibit CAC activities that are concordant with the mitochondrial activity of the host cells.

Interestingly, LDH activity was also detected in isolated mitochondrial suspensions; hence, we were able to compare the isotopic enrichments for lactate between mitochondria from NT2196 cells and muscle tissues. In agreement with the greater dependence of HER2+ cancer cells on aerobic glycolysis, mitochondria isolated from NT2196 cells displayed drastically greater lactate m + 3 than those from muscle tissues (Figure 4F). It is important to emphasize that the enzyme, LDH, has been shown to be associated with mitochondria [39]. Other enzymes, such as glucokinase, have also been shown to be associated with mitochondrial membranes [45]. Although we cannot exclude the possibility that LDH activity in our experiments is partly coming from contaminating cytoplasmic LDH during the isolation procedure, the finding that LDH-associated activity can exhibit profound differences in activity according to the mitochondrial source is of great bioenergetic interest.

Overall, the data presented in this manuscript reveal that the assessment of carbon flow in isolated mitochondria can be useful for determining whether changes in metabolism at the cellular level in physiological or pathological conditions are associated with modulation in the intrinsic activities of mitochondria. The methodology presented here used U-^13^C-pyruvate and unlabeled malate as substrates; however, other combinations could be used to assess other metabolic activities of mitochondria. In addition, the usage of ^13^C-glutamate would prove useful for the assessment of reductive carboxylation in isolated mitochondria.

## 3. Experimental Section

### 3.1. Animals, Cells and Reagents

Wild-type male C57 black 6 Inbr (J) mice (C57BL/6J, stock no. 000664) were purchased from The Jackson Laboratory (Bar Harbor, ME, USA). NT2196 cells were kindly provided by William Muller (McGill University, Montreal, Canada) and have been described elsewhere [43]. All reagents and mitochondrial inhibitors were purchased from Sigma-Aldrich, unless otherwise stated.

### 3.2. Isolation of Mitochondria from Mice

Mice were sacrificed at approximately 6 months of age in accordance with the animal protocols from the McGill University Animal Care Committee. Mitochondria from skeletal muscle were isolated as previously described [46].

### 3.3. Isolation of Mitochondria from Cultured Cells

NT2196 cells were cultured in 15-cm plastic dishes in a humidified incubator set at 37 °C and 5% CO_2_, as previously described [42]. Cells at 90% confluence were scraped on ice, washed with phosphate buffer saline (PBS), harvested by centrifugation at 850× g for 5 min and resuspended in 5 volumes of Buffer A (250 mM sucrose, 10 mM KCl, 1 mM EDTA (ethylenediaminetetraacetic acid), 1mM EGTA (ethylene glycol tetraacetic acid), 1.5 mM MgCl_2_, 20 mM HEPES (4-(2-hydroxyethyl)-1-piperazineethanesulfonic acid), 1% BSA (bovine serum albumin fraction V) (w/v), pH 7.4) [47]. The following steps were performed at 4 °C. Cells were homogenized with a Wheaton glass pestle until approximately 80% of cell lysis was confirmed under a light microscope. The suspension was centrifuged at 1000× g for 7 min, and the supernatant was re-spun at 1000× g for 7 min and then again at 10,000× g for 15 min. The pellet was washed in 2 mL of Buffer A and spun again at 10,000× g for 15 min. The resulting pellet was dissolved in mitochondrial assay buffer (KHEB buffer: 120 mM KCl, 5 mM KH_2_PO_4_, 3 mM HEPES, 1 mM EGTA and 0.3% BSA (w/v), pH 7.2) [46]. Mitochondrial proteins were quantified using a standard Bradford assay with 1:3 Bradford dye reagent (Bio-Rad).

### 3.4. Respiration

Respiration measurements with mitochondria isolated from skeletal muscle, as well as from cultured cells were performed using a Digital Model 10 Clark Electrode (Rank Brothers, Cambridge, UK). Isolated mitochondria were incubated in KHEB buffer (Section 3.3) at 0.6 mg mitochondrial proteins per millilitre at 37 °C. Respiratory control ratios (RCRs) were used to determine the quality of the mitochondrial suspensions. RCR values were obtained by dividing the rate of oxygen consumption in the presence of ADP (State 3) by that in the presence of oligomycin (State 4). Only mitochondrial suspensions with RCR values greater than 3 were used for further studies.

### 3.5. Extraction of Metabolites from Cells and Isolated Mitochondria

NT2196 cells were grown in 35-mm plates to 70%–80% confluency, washed once with sterile PBS and then incubated for 1 h in DMEM (Dulbecco’s Modified Eagle’s medium) high glucose media supplemented with 10% dialyzed FBS (Wisent), 20 mM HEPES, 10 µg/mL insulin, 4 mM L-glutamine and 10 mM U-^13^C-D-glucose (Cambridge Isotope Laboratories, CLM-1396, 99% atom ^13^C). The medium was then aspirated; the cells were rinsed once with 2 mL saline solution (9 g/L NaCl, 4 °C) and then overlaid with 300 µL of 80% MeOH solution kept on dry ice. Cells were scraped twice in this solution, and extracts were transferred into pre-chilled 1.5-mL tubes. Tubes were vortexed and stored at −80 °C until further treatments.

Isolated mitochondria, corresponding to 0.15 mg mitochondrial protein (Section 3.3), were resuspended in a modified KHEB buffer with low phosphate content (120 mM KCl, 0.2 mM KH_2_PO_4_, 1 mM HEPES, 0.5 mM EGTA and 0.3% BSA (w/v), pH 7.2). Labeled or unlabeled substrates (1 mM pyruvate or 1 mM U-^13^C-pyruvate (Sigma, 490717, 99% atom ^13^C); 1 mM malate) were added to the mitochondrial suspensions. The final incubation volume was 100 µL. Time 0 of the pulse experiments was set when mitochondrial suspensions were placed in a water bath maintained at 37 °C with mild agitation in the presence of either rotenone (0.05 mM), antimycin (0.05 mM) or an equivalent volume of DMSO. Reactions were stopped by adding 400 µL of 100% MeOH maintained on dry ice (a final concentration of 80% MeOH). Tubes were rapidly vortexed and then maintained on dry ice for 10 min before being stored at −80 °C.

### 3.6. GC/MS

Prepared samples (Section 3.5) were sonicated and cleared by centrifugation. Supernatants were dried overnight in a cold centri-trap (Labconco). Pellets were resuspended in 30 µL methoxyamine hydrochloride dissolved in anhydrous pyridine (Sigma), vortexed, sonicated, centrifuged and incubated at 70 °C for 30 min in sealed-cap injection tubes. Seventy microliters of MTBSTFA (Sigma, 375934) were added to the supernatants, and the samples were heated at 70 °C for 60 min. GC/MS injections and analyses were performed on Agilent instruments and software, as previously described [48].

### 3.7. Mass Isotopomer Distribution Analysis

The integration of peak areas for specific ions (see Table 2) analyzed in SIM mode was done with the ChemStation software (Agilent). These ions represent, from left to right, m + k, where m is the molecular weight of the principal quantifying fragment, –57 (C_4_H_9_), and k is the number of additional atomic mass units (k:0 → *n*, where *n* is the maximal amount of labeled carbons). The amount of a given metabolite in a sample was estimated with the sum of integrations over all associated ions, Σ (m + k), divided by the integration of the m/z 312 ion monitored for the internal standard D_27_-myristic acid. This value is termed “corrected area”. For mitochondrial stable isotope tracer analysis, the latter ratio was not further corrected for mitochondrial protein content, since this quantity is constant for each independent sample (0.15 mg).

**Table 2:** List of metabolites monitored by GC/MS and fragments used for SIM.

Metabolite	Molecular formula	m/z quantifying ions (qualifyer)	Retention time (min)
Pyruvate oxime	C_6_H_12_NO_3_Si	174–177 (115)	8.978
Lactate	C_11_H_25_O_3_Si_2_	261–264 (233)	11.742
Alanine	C_11_H_26_NO_2_Si_2_	260–263 (232)	12.312
Succinate	C_12_H_25_O_4_Si_2_	289–293 (331)	14.813
Fumarate	C_12_H_23_O_4_Si_2_	287–291 (329)	15.208
Oxaloacetate oxime	C_13_H_26_NO_5_Si_2_	332–336 (374)	16.068
Serine	C_17_H_40_NO_3_Si_3_	390–393 (362)	17.108
Alpha-ketoglutarate oxime	C_14_H_28_NO_5_Si_2_	346–351 (258)	17.319
Malate	C_18_H_39_O_5_Si_3_	419–423 (287)	18.275
Aspartate	C_18_H_40_NO_4_Si_3_	418–422 (390)	18.638
Glutamate	C_19_H_42_NO_4_Si_3_	432–437 (330)	19.685
Citrate	C_20_H_39_O_6_Si_3_	459–465 (431,403)	22.429
Isocitrate	C_20_H_39_O_6_Si_3_	459–465 (431,403)	22.518
D_27_-myristic acid	C_16_H_6_D_27_O_2_Si	312 (132)	17.912

Mass isotopomer distribution analyses were performed using a method adapted from [49]. This mathematical procedure was applied to each metabolite analyzed in order to remove the contribution of natural isotopes (^2^H, ^3^H, ^13^C, *etc.*) to the monitored ion integrations and, thus, allowing the exclusive analysis of exogenous ^13^C contribution provided by U-^13^C-glucose (in cells) or U-^13^C-pyruvate (in isolated mitochondria). Briefly, mass distribution vectors α (MDVα) grouping all integrations values for m + k for a given metabolite in a sample were multiplied by metabolite-specific corrections matrices (generated for TBDMS (tert-butyldimethylsilyl)-derivatized M-57 fragments using an in-house algorithm) to generate mass distribution vectors corrected for natural isotopes abundances. With elements expressed as a fraction of 1, we name this vector mass isotopomer distribution (MID). The values obtained for each m + k represent the isotopomer proportions of individual ions within the pool of a given metabolite for each sample. This value does not give information about the total amount of a given metabolite present in a sample. Mass isotopomer distribution analysis was used to determine the contribution of ^13^C-glucose into glycolytic and CAC metabolites in cultured cells. The assay was not designed to assess the isotopic steady state of metabolites (saturated contributions from ^13^C), but to delineate the contribution of glucose to glycolysis and CAC at a single time point (1 h) that was determined to provide sufficient labeling of CAC intermediates for reproducible measurements. For stable isotope tracer analysis in isolated mitochondria from muscle and cultured cells, MID multiplied by corrected area (MID × corrected area) gives information on both the amount of a given metabolite in a sample and its isotopomer composition.

### 3.8. Statistical Analyses

Statistical analyses were performed with Microsoft Excel and GraphPad Prism. Results are presented as means ± SEM from 3 independent experiments, unless specified. The statistical significance threshold was set at a *p*-value of 0.05.

## 4. Conclusions

Altered metabolism is a hallmark of cancer cells [40]. The emergence of metabolomics in cancer research has allowed for the identification of metabolic signatures unique to specific cancers [50]. Furthermore, metabolomics provides a new approach for target identification and validation. The methodology presented in this manuscript demonstrates that metabolomics studies can be performed on isolated organelles from tissues or cultured cells. We have shown that the activity of the CAC is altered in isolated mitochondria when they are treated with specific ETC inhibitors or when mitochondria are isolated from cells or tissues displaying different metabolic properties. Overall, the methodology presented here will be useful for direct metabolomics analyses of organelles in physiological or pathological conditions.
